# Grey Matter Changes Associated with Heavy Cannabis Use: A Longitudinal sMRI Study

**DOI:** 10.1371/journal.pone.0152482

**Published:** 2016-05-25

**Authors:** Laura Koenders, Janna Cousijn, Wilhelmina A. M. Vingerhoets, Wim van den Brink, Reinout W. Wiers, Carin J. Meijer, Marise W. J. Machielsen, Dick J. Veltman, Anneke E. Goudriaan, Lieuwe de Haan

**Affiliations:** 1 Department of Psychiatry, Academic Medical Centre, University of Amsterdam, Amsterdam, The Netherlands; 2 Department of Nuclear Medicine, Academic Medical Centre, University of Amsterdam, Amsterdam, The Netherlands; 3 Department of Mental Health and Neuroscience, Maastricht University, Maastricht, The Netherlands; 4 Addiction Development and Psychopathology (ADAPT)-lab, Department of Developmental Psychology, University of Amsterdam, Amsterdam, The Netherlands; 5 Cognitive Science Center Amsterdam, University of Amsterdam, Amsterdam, The Netherlands; 6 Department of Developmental Psychology and Psychonomics, Utrecht University, Utrecht, The Netherlands; 7 Arkin Mental Health Care, Amsterdam, The Netherlands; 8 University Medical Center, Vrije Universiteit Amsterdam, Amsterdam, The Netherlands; Banner Alzheimer's Institute, UNITED STATES

## Abstract

Cannabis is the most frequently used illicit drug worldwide. Cross-sectional neuroimaging studies suggest that chronic cannabis exposure and the development of cannabis use disorders may affect brain morphology. However, cross-sectional studies cannot make a conclusive distinction between cause and consequence and longitudinal neuroimaging studies are lacking. In this prospective study we investigate whether continued cannabis use and higher levels of cannabis exposure in young adults are associated with grey matter reductions. Heavy cannabis users (N = 20, age baseline M = 20.5, SD = 2.1) and non-cannabis using healthy controls (N = 22, age baseline M = 21.6, SD = 2.45) underwent a comprehensive psychological assessment and a T1- structural MRI scan at baseline and 3 years follow-up. Grey matter volumes (orbitofrontal cortex, anterior cingulate cortex, insula, striatum, thalamus, amygdala, hippocampus and cerebellum) were estimated using the software package SPM (VBM-8 module). Continued cannabis use did not have an effect on GM volume change at follow-up. Cross-sectional analyses at baseline and follow-up revealed consistent negative correlations between cannabis related problems and cannabis use (in grams) and regional GM volume of the left hippocampus, amygdala and superior temporal gyrus. These results suggests that small GM volumes in the medial temporal lobe are a risk factor for heavy cannabis use or that the effect of cannabis on GM reductions is limited to adolescence with no further damage of continued use after early adulthood. Long-term prospective studies starting in early adolescence are needed to reach final conclusions.

## Introduction

Cannabis is the most frequently used illicit drug worldwide [1]. The use of cannabis has been associated with an increased risk of mood and anxiety disorders [2–4], psychotic symptoms and psychosis [5–8], and with cognitive impairment [9]. Furthermore, cross-sectional neuroimaging studies suggest that chronic cannabis exposure and the development of cannabis use disorders may affect brain morphology [10, 11]. For example, several cross-sectional studies have shown that regular cannabis use is associated with smaller grey matter (GM) volumes in medial temporal regions (e.g. hippocampus and amygdala) and parts of the prefrontal cortex, and with increased GM volume in the cerebellum [10–13]. Yet, conflicting results have also been reported, with several studies showing no morphological brain differences between regular cannabis users and non-cannabis using controls [14–20], and some others reporting even larger regional brain volumes in cannabis users compared to controls [12, 17, 21]. These conflicting results may be related to differences in cannabis exposure and/or differences in severity of cannabis dependence across studies, which are reported to be potential confounding factors [11, 22]. Our previous study concerning the baseline assessment of the current sample [17] showed that amygdala volume was inversely correlated with cannabis related problems and that hippocampal volumes were inversely correlated with average weekly cannabis use (gram). In addition, we found that cerebellar volumes were larger in cannabis users than in controls. Besides these findings no other associations were found between brain volumes and cannabis use or cannabis related problems [17]. For an overview of the findings of our baseline paper and the current paper, see Table A in S1 Table.

However, none of these cross-sectional studies allow a conclusive distinction between the role of cannabis use as either a cause or a consequence of brain abnormalities, and longitudinal neuroimaging studies are lacking. We therefore conducted a three-year longitudinal structural neuroimaging study in a group of long-term young adult heavy cannabis users (CB) and a matched group of non-cannabis using healthy controls (HC). The aim of the study was twofold: first, to examine whether GM changed over time in the CB group compared to the HC group; and second, to investigate within the CB group the association between change in regional GM volumes and change in severity of cannabis use related problems or levels of cannabis exposure.

Based on our baseline findings (GM volume in the anterior cerebellum larger in the CB than in the HC group, and negative correlations of cannabis exposure (gram per week) and cannabis related problems and GM volumes in the hippocampus and amygdala respectively within the CB group [17]) and on findings from previous structural neuroimaging findings, we hypothesized reductions in GM volumes in the CB group compared to the stable volumes in the HC group in the orbitofrontal cortex (OFC) [23], anterior cingulate cortex (ACC) [24], insula [25], striatum [24], thalamus [24], amygdala [24], hippocampus [22] and cerebellum [17]. In addition, within the CB group we expected that changes in GM volume over time would be related to changes In cannabis use related problems and cannabis exposure in these regions of interest.

## Materials and Methods

### Participants

This study was part of a three-year longitudinal examination of cannabis users [26]. Participants were assessed at baseline (BL, 2009) and follow-up (FU, 2012). The mean time between baseline and follow-up was 39 months (SD = 2.4 months). Of the 33 heavy CB users and 43 non-drug using HCs recruited at baseline, 24 CB subjects (73%) and 27 HC subjects (63%) completed the FU session. The remaining 25 subjects did not participate in the FU assessment for different reasons: contact lost (N = 12), refused (N = 10), or not available (N = 3). We excluded nine participants: low scan quality (N = 3), CB subjects that stopped using cannabis (N = 3) and HC subjects that started to use cannabis or other drugs (N = 3), leaving 20 persistent CB users and 22 persistent HCs for analysis.

Recruitment was done through advertisements on the Internet and in cannabis outlets (coffee-shops). All participants were instructed to abstain from alcohol and drugs 24 hours before the assessments. At baseline, heavy cannabis use was defined as using cannabis for at least two years, more than 10 days per month and not seeking treatment or having a history of treatment for cannabis use. Participants in the control group used cannabis fewer than 30 times ever in their life and did not use last year. For more detailed information on participant inclusion and exclusion criteria, see Cousijn et al. [17].

At both time assessments, all participants underwent MRI scanning, and a detailed history of cannabis use and a comprehensive battery of questionnaires was administered. Urine samples were taken prior to both test sessions to detect recent illicit drug use. Although urine analysis of Δ9-tetrahydrocannabinol (THC) is not suitable for the detection of 24-hour cannabis abstinence, it increases accuracy of self-reported substance use [27].

The medical ethics committee of the Academic Medical Centre approved the study and all participants signed informed consent before participation after the study procedure had been explained.

### Materials

Demographic information was collected at baseline and follow-up. Severity of cannabis use was measured using the Cannabis Use Disorder Identification Test [28]. The CUDIT is a screening instrument for at risk problematic cannabis use and consists of 10 items on cannabis use frequency and on severity of use-related problems. All participants were questioned about their history of cannabis use, from which we derived their weekly cannabis use in grams. At baseline, we did not set a time window for ‘average cannabis use (gram)’ but left the period open to the interpretation of the participant. At follow-up, we specified the time window was the three years in between the two assessments. The severity of nicotine dependence was assessed using the Fagerström Tolerance Questionnaire [29]. To assess (problem) severity of alcohol use we administered the Alcohol Use Disorder Identification test [30]. Premorbid intellectual functioning was estimated by the Dutch Adult Reading Test [31]. At follow-up, the Mini International Neuropsychiatric Interview [32] was conducted by two experienced psychologists to assess the prevalence of mental disorders according to DSM-IV.

### MRI acquisition and processing

All structural MRI scans were acquired using a 3T MRI scanner (Intera, Philips Healthcare, Best, The Netherlands) with a phased array SENSE eight-channel receiver head coil. For each participant, a T1-weighted structural MRI image was acquired (T1 turbo field echo, TR 9.6 s, TE 4.6 s, 182 slices, slice thickness 1.2 mm, FOV 256x256 mm, in-plane resolution 256x256 mm, flip angle 8°). T1-weighted images were visually inspected for motion and other artifacts. After quality evaluation of the data, we manually reoriented the images to the anterior commissure.

For both the cross-sectional and the longitudinal analyses, we used the VBM-8 module of the software package SPM (Structural Brain Mapping Group, University of Jena, Germany, http://dbm.neuro.uni-jena.de/vbm8). For the cross-sectional analyses, at baseline and follow-up, individual T1 images were first aligned to a T1 template in MNI-space (Montreal Neurological Institute) and subsequently segmented into grey matter, white matter and cerebro-spinal fluid. The resulting grey matter was normalized using the diffeomorphic image registration algorithm (DARTEL) [33]. Grey matter images were modulated with the nonlinear transformation parameters as computed during the normalization procedures. The resulting images contain the volume proportion of probabilistically assigned grey matter tissue for each voxel. These grey matter tissue probability images were visually inspected again and finally smoothed with an 8-mm Gaussian kernel. Note that each image of the regional grey matter volume was corrected for individual brain size, since this step is part of the VBM8 toolbox routine.

For the longitudinal analyses, MRI data processing was performed using the VBM8 longitudinal batch, which has specific preprocessing steps for longitudinal data. These steps are summarized as follows: (1) registration of the follow-up image to the baseline image for each participant; (2) calculation of the mean image from the realigned images for each participant. This mean image is used as a reference image for subsequent spatial alignment; (3) the realigned images are corrected for field inhomogeneities with regard to the reference mean image; (4) tissue segmentation is performed in the bias-corrected mean reference image and the bias-corrected realigned images; (5) DARTEL spatial normalization parameters are estimated using the tissue segments (grey matter and white matter) of the bias-corrected mean reference image; (6) normalization parameters are applied to the tissue segments of the bias-corrected realigned images; and (7) the resulting normalized tissue segments for each time point of each participant are smoothed with an 8-mm Gaussian kernel. To avoid possible edge effects between grey and white matter, all voxels with grey matter values <0.1 were excluded (absolute threshold masking).

Finally, to assess the relation between GM change over time and change in respectively CUDIT scores and amount of grams per week, GM difference images were calculated with ImCalc by subtracting each subject’s probability map at baseline from the map at follow-up.

ROIs were based on our previous cross-sectional baseline study (OFC, ACC, insula, striatum, thalamus, amygdala, hippocampus and cerebellum) and were anatomically defined using the Nielsen and Hansen’s volume of interest online database [34]. The results are visualized using xjView toolbox (http://www.alivelearn.net/xjview).

### Data analyses

Demographic data were compared between groups and over time using repeated measures analysis of variance tests, independent samples t-tests and paired samples t-tests.

To assess cross-sectional group differences in GM volume we performed two-sample t-tests comparing CBs and HCs at baseline and follow-up separately. In addition, to assess if grey regional grey matter volume changed over time in the CB group compared to the HC group, a full factorial model was used. Of note, even though the same scan protocol was used at baseline and follow-up, intermittent scanner updates introduced time-dependent changes in the MRI signal (independently of group). Therefore only group by time interaction effects can be reliably reported.

To investigate cross-sectional relations between cannabis use-related problems (CUDIT) and grams per week with GM volume, we performed multiple regression analyses within the CB group, also at baseline and follow-up separately. Subsequently, we studied the relation between GM change over time and change in CUDIT scores and grams per week.

To check the assumptions of the analyses, we checked for normality. GM was normally distributed in all five significant ROIs. Regarding the independent variables, only weekly amount of use (gram) measured at follow-up deviated from normality. We transformed this variable using the method described in Osborne [35] for a control analysis. Age and gender were included as covariates in all analyses. In addition, we assessed the potential confounding effects of comorbid nicotine use, alcohol dependence, other drug use and/or psychiatric disorders; we repeated the analyses separately with respectively FTQ scores, cigarettes per week, AUDIT scores and frequency of alcohol use as covariates. In addition, we repeated the analyses with exclusion of the subjects with comorbid substance use disorders (other than cannabis use disorders) and/or psychiatric disorders (leaving 14 cannabis users and 20 healthy controls).

For all analyses, we first performed region of interest (ROI) analyses, where structural differences within ROI masks were considered significant if *p* < .001, with an FWE-corrected cluster probability of *p* < .05 adjusted for the small search volume [36]. In addition, we performed an exploratory whole brain analysis, where structural differences outside ROI masks were considered significant if *p* < .001, with a whole brain FWE-corrected cluster probability of *p* < .05.

## Results

### Sample characteristics

For demographic variables see Table 1. As expected, CB subjects showed significantly higher cannabis and nicotine dependence scores than HCs, reflected by respectively higher CUDIT and FTQ scores for the CB compared to the HC group at both time points. CUDIT scores were stable over time, but FTQ scores were higher at follow-up for both groups. All but one subject in the CB group smoked their cannabis with tobacco. In the HC group, 2 subjects used cannabis 20–25 times in their lifetime, the rest used cannabis less than 5 times. At baseline 3 HC subjects (14%) were smoking tobacco, and at follow-up 3 had started and 1 had stopped smoking. Alcohol problem severity (AUDIT scores) increased over time but changes did not significantly differ between groups. In addition, we have looked at the group difference in alcohol consumed. There was no difference in consumed alcohol at baseline (χ^2^(4) = 5.41 p = .23). However, the difference between cannabis users and controls was significant at follow-up (χ^2^(4) = 11.79, p = .02).

**Table 1:** Demographic information in mean and standard deviations, per group (heavy cannabis users, CB and healthy controls, HC) per time point (baseline and follow-up).

	CB (N = 20)		HC (N = 22)		Statistics	
	Baseline	Follow-up	Baseline	Follow-up	Change over time	Group difference
**Gender, % male**	75		64			χ^2^(1) = .63, *p* = .43
**Age, yrs**	20.5 (2.11)	24.0 (2.48)	21.6 (2.45)	24.8 (2.42)	***F* = 791.46, *p* < .001**	*F* = 1.53, *p* = .22
**DART**	104 (5)	105 (5)	106 (6)	103 (9)	*F* = .70, *p* = .41	*F* = .07, *p* = .78
**CUDIT**	12.70 (6.59)	13.25 (8.31)	.05 (0.21)	.18 (.39)	*F* = .22, *p* = .65	***F* = 82.40, *p* < .001**
**AUDIT**	6.25 (3.35)	8.50 (4.96)	4.41 (3.38)	6.18 (3.45)	***F* = 13.96, *p* = .001**	*F* = 3.91, *p* = .06
**Alcohol use, frequency**^**a**^						
**no or sporadic drinking**	4	3	10	1		
**Less than weekly, light drinking**	4	0	5	8		
**Weekly, light drinking**	8	4	3	5		
**Weekly, heavier drinking**	3	11	2	5		
**Weekly, heavy drinking**	1	2	2	3		
**FTQ**	2.75 (2.40)	4.93 (1.9)	.41 (1.05)	3.20 (1.64)	***F =* 20.05, *p* < .001**	***F* = 4.86, *p* = .04**^**#**^
**cigarettes/day**^b^^*^	7.08 (7.37)	11.87 (8.57)	1.14 (3.06)	5.70 (10.81)	*F* = 1.14, *p* = .30	*F* = 3.38, *p* = .08
**Cannabis use**						
**onset first use, age yrs**^**c**^	14.50 (1.65)		18.46 (2.99)		NA	**t(17) = 4.37, *p* < .001**
**onset regular use, age yrs**	16.29 (2.35)				NA	NA
**current use, gr/week**	2.78 (1.78)	3.47 (3.26)			t(19) = -.95, *p* = .35	NA
**current use, days/week**	4.70 (1.62)	5.10 (2.34)			t(19) = -.87, *p* = .40	NA

There were no time by group interaction effects. The statistics are reported for the total group (change over time for CB and HC) and a total for both time points (group difference on BL and FU). Significant results are printed in bold. Note: CB = cannabis group; HC = healthy control group; DART = Dutch Adult Reading Test; CUDIT = Cannabis Use Disorder Identification Test; AUDIT = Alcohol Use Disorder Identification Test; FTQ = Fagerström Tolerance Questionnaire

^a^ light drinking: 1–4 units, heavier drinking: 5–9 units, heavy drinking: >5 units, more than 2 times a week

^b^ CB group N = 14, HC group N = 5

^c^ CB group N = 20, HC group N = 13

* all but one CB users smoked their cannabis with tobacco

^#^ significant difference on BL (p = .04) but not on FU (p = .09).

Participants had several comorbid DSM-IV diagnoses, which were assessed at follow-up. In the CB group, there were 4 participants (20%) with an alcohol use disorder diagnosis and 3 participants (15%) with multiple substance abuse dependence diagnoses; i.e. cocaine and amphetamine use. In addition, there was one participant (5%) with a psychotic disorder, one participant (5%) with an affective disorder and 3 participants (15%) with multiple psychiatric diagnoses; e.g. a combination of ADHD and an anxiety or an affective disorder. In the HC group 3 participants (14%) developed an alcohol use disorder diagnosis and 1 participant (5%) became dependent on both cocaine and amphetamine. In addition, 1 HC (5%) developed an affective disorder. Due to subjects with multiple diagnoses, this left a total of 14 cannabis users and 20 healthy controls.

### GM volume group comparisons

Our previously reported cross-sectional finding of a cluster with larger grey matter volume in the CB group compared to the HC group in the cerebellum [17] is still significant in the present sample at baseline (cluster size = 393, p_FWE_ = .035, x,y,z = 26,-70,-20). However, this difference was no longer significant at follow-up (p_FWE_ = n.s.). Also, this cluster was no longer significant after inclusion of any of the control covariates except frequency of alcohol use (cluster size = 466, p_FWE_ = .023, x,y,z = 26,-58,-20 and without subjects with comorbid substance or psychiatric disorders (p_FWE_ = n.s.; Table B in S1 Table). There were no group differences in the other ROIs or at whole brain level at both baseline and follow-up.

To assess whether regional grey matter volume showed different changes over time in the CB-group compared to the HC-group, we looked at the interaction term between group and time. There were no significant interactions in any of the ROIs or at whole-brain level, indicating that grey matter volume changes were similar in the two groups.

### GM volume associations with cannabis use characteristics

**Fig 1:**
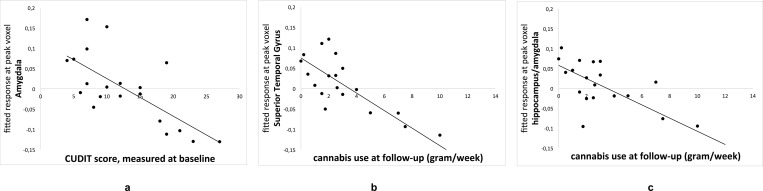
Correlation between the GM density at the peak voxel per ROI and the independent variable. Fig 1a: correlation between CUDIT score at baseline and GM density in the amygdala. Fig 1b: correlation between average weekly cannabis use (gram) at follow-up and GM density in the Superior Temporal Gyrus. Fig 1c: correlation between average weekly cannabis use (gram) at follow-up and GM density in the Medial Temporal Lobe (hippocampus/amygdala).

**Fig 2:**
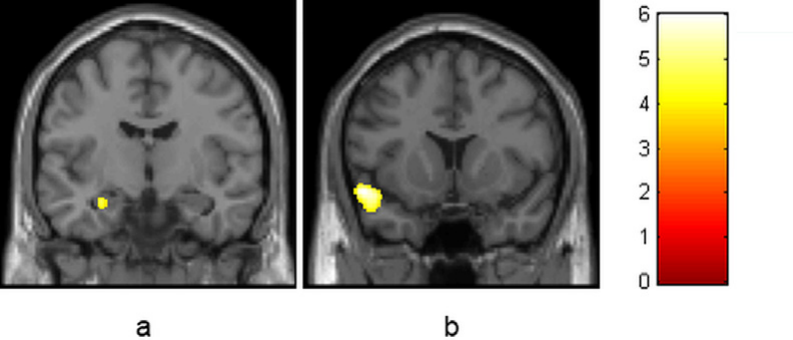
Negative correlation between use per week in grams at follow-up and grey matter volume in the whole brain analysis in the CB group. There are two significant clusters, one in the hippocampus/amygdala (cluster size = 118, pFWE = .044, x,y,z = -27,-10,-20; Fig 2a) and one in the superior temporal gyrus (cluster size = 518, pFWE = .044, x,y,z = -52,11,-15; Fig 2b). Images are coronally depicted in radiological convention (i.e. subject’s left is image’s right).

Our previously reported finding of an inverse correlation between amygdala volume and cannabis related problems [17] is still significant in the current sample. At baseline, there was a significant negative correlation between CUDIT score and grey matter volume in a cluster in the left amygdala (cluster size = 8, p_FWE_ = .034, x,y,z = 22,-4,-23; Fig 1A). This cluster was no longer significant after inclusion of any of the control covariates except frequency of alcohol use (remaining cluster size = 3, p_FWE_ = .039) and without subjects with comorbid substance or psychiatric disorders (p_FWE_ = n.s.; Table B in S1 Table). This cluster was also no longer significant at follow-up, but there was a significant negative correlation between the amount of use in grams per week and grey matter volume in a cluster comprising the amygdala (cluster size = 43, p_FWE_ = .018, x,y,z = -28,-9,-21) and hippocampus (cluster size = 118, p_FWE_ = .044, x,y,z = -27,-10,-20, Figs 1C and 2A) at follow-up. This cluster remained significant after controlling for severity of nicotine use (remaining cluster size = 55, p_FWE_ = .015), frequency of nicotine use (remaining cluster size = 20, p_FWE_ = .027), severity of alcohol use (remaining cluster size = 28, p_FWE_ = .023), frequency of alcohol use (remaining cluster size = 31, p_FWE_ = .022), and without subjects with comorbid substance or psychiatric disorders (remaining cluster size = 8, p_FWE_ = .039; Table B in S1 Table). Exploratory whole-brain analyses showed a significant negative correlation between amount of use in grams per week and grey matter volume in a cluster in the superior temporal gyrus (cluster size = 518, p_FWE_ = .044, x,y,z = -52,11,-15; Figs 1B and 2B). This cluster was no longer significant after inclusion of any of the covariates (Table B in S1 Table).

To assess whether change in GM volumes were related to change in problems related to cannabis use, linear regression analyses were performed with change in regional GM volume as dependent variable and change in CUDIT score and change in cannabis use (grams per week) as independent variables. We found no significant correlations between the CUDIT score and cannabis use measures and any of the ROIs or whole-brain analyses.

## Discussion

The current 3-year longitudinal study investigated the relation between heavy cannabis use and regional grey matter volume over time. GM volumes in the OFC, ACC, insula, striatum, thalamus, amygdala, hippocampus and cerebellum were prospectively compared between a CB and HC group and related to cannabis use measures within the CB group. In contrast to our hypothesis, there were no differences between the CB and the HC group in GM volume changes. Moreover, changes in weekly cannabis use and cannabis use related problems were not significantly related to changes in GM volumes. Finally, cross-sectional analyses at baseline and follow-up revealed consistent correlations between GM volumes in the left medial temporal lobe (i.e., hippocampus, amygdala, STG) and cannabis related problems and cannabis use (in grams). Our previously reported finding of an inverse relationship of hippocampal volume and amount of use (gram/week) is not significant in the current (smaller) sample. Interestingly, we do find this relationship at follow-up. In addition, our finding of larger cerebellar volumes in cannabis users than controls (found in the larger sample of 33 cannabis users and in the current (sub)sample at baseline is no longer significant at follow-up (for an overview of the findings of our baseline paper and the current paper, see Table A in S1 Table). During typical development, cerebellar volumes decrease over time [37]. Possibly, in cannabis users, this decrease is occurring at a later age, which may explain the group difference observed at baseline but not at follow-up. However, we cannot confirm this hypothesis with only two assessments, and future studies should take this into account.

The lack of significant changes between groups in GM volume over time suggests that cannabis use (>5 years) during late adolescence and early adulthood does not change GM morphology. The cross-sectional negative correlations between medial temporal lobe volumes and the severity of cannabis use suggests that cannabis use related changes in GM were already present before the onset of our study and did not worsen after continued use in adolescence. On the other hand, we cannot rule out that these GM correlations may have been already present before the initiation of cannabis use and may represent a vulnerability factor for heavy use or dependence.

If the cross-sectional relations in temporal brain areas are a direct consequence of (heavy) cannabis use, this means that only those with the most severe patterns of cannabis use will suffer from GM volume reductions but that these GM reductions do not get worse with continued heavy cannabis use after a certain age, i.e. in our sample—with a mean age of 20 years—the maximum volume decreases would have already been reached. This interpretation of our cross-sectional findings is in line with recent conclusions from two reviews [11, 38] suggesting that smaller GM volumes in cannabis users are mostly present in temporal brain areas, but may only be evident in the most severe users. Correlations between the amount of use and medial temporal lobe volumes have been described before [17, 39, 40], and abnormalities in the hippocampus and amygdala have consistently been found in (heavy) cannabis users [11, 22]. Both the amygdala [41, 42] and the hippocampal region [43] contain high levels of cannabinoid receptors making these regions likely to be affected by heavy as opposed to incidental cannabis use.

The lack of longitudinal associations between (changes in) cannabis use and changes in GM volumes over time, however, makes it unlikely that our cross-sectional differences in GM volumes are the result of heavy cannabis use. Another explanation is therefore that our cross-sectional findings represent a vulnerability factor for the development of cannabis use related problems (including the inability to control the amount of use). Cheetham et al. (2012) found that smaller orbitofrontal cortices at age 12 predicted initiation of cannabis use by age 16, but temporal regions did not predict later cannabis use [44]. On the other hand, in alcohol users, smaller temporal lobe volumes have shown to be a risk factor for alcohol abuse, rather than a consequence [45, 46]. This may imply that for substances like alcohol and cannabis, regional brain volume may be indicative for later substance use, and thus a pre-existing risk factor. This interpretation is further supported by our finding that the negative correlation between the medial temporal lobe GM volume and cannabis related problems was dependent on the severity of both alcohol and nicotine dependence. This may imply that a small medial temporal lobe is a vulnerability factor for substance dependence in general. The amygdala is involved in drug reward processing and the avoidance of drug abstinence [24], as well as in cue-elicited craving [47], whereas the hippocampus is supposedly involved in the learning of affective states in relationship to drug intake [48]. Structural deviations in these regions could render a subject more vulnerable to the development of substance dependence. Future studies can hopefully provide more insight in this issue by including non-using relatives or using prospective designs studying subjects before the onset of cannabis use. The Adolescent Brain Cognition Study from the Collaborative Research on Addiction at NIH (CRAN; http://addictionresearch.nih.gov/) for example might provide an opportunity to disentangle these different complicated relationships between GM development and cannabis use.

### Limitations

The longitudinal nature of this study is its main strength, but some limitations have to be considered. First, our participants used alcohol, tobacco, and developed DSM-IV diagnoses over time. The CB group used more nicotine than the HCs, since all CB users but one smoked their cannabis with tobacco. In addition, several participants developed DSM-IV diagnoses over time. Regarding the severity of alcohol use, there was no difference between the groups, making it unlikely that the lack of effect over time is influenced by concomitant alcohol use. Nevertheless, we controlled for these nuisance factors. The negative correlation between the amount of cannabis use and the medial temporal lobe GM volumes at follow-up remained significant. However, other cross-sectional results were no longer significant. It is possible that the reduction in power lead to a loss of significance in our other results, but it cannot be ruled out that effects of nicotine, severity of alcohol use or comorbid psychiatric disorders did contribute to the group differences we observed. To further assess the relation between cannabis, nicotine and alcohol use and dependence, future studies should include healthy, nicotine and/or alcohol dependent controls. To account for comorbid psychiatric disorders, future longitudinal studies should assess psychiatric symptoms and include a large enough sample to have sufficient power to control for these nuisance factors. Second, the cannabis users were young, otherwise healthy cannabis users using substantial levels of cannabis (using on average 5 days a week for 5 years). However, they showed steady levels of cannabis use over at least 5 years, suggesting a substantial but relatively controlled level of use. Several studies investigating samples with comparable use had negative results [14, 20, 21]. However, other studies with at least partly comparable samples did report group differences between cannabis users and controls [12, 15, 49, 50]. Therefore, we think that the negative prospective findings are not due to the relatively controlled (but still very substantial) levels of cannabis use in our sample. Furthermore, future studies should explore the differences in cannabis use measures. Our results showed an inverse relationship between amygdala volumes and the CUDIT at baseline, and between the amygdala and amount of use at follow-up. These results may be explained by overlapping variance in cannabis use and the level of use related problems, since the CUDIT contains questions regarding amount of cannabis use. However, individuals using the same amount may experience different levels of problems. In a future study with a larger sample, we expect significant relations with both cannabis use measures. In addition, our baseline measure of weekly average amount of use (gram) was left to the interpretation of the user, since we did not specify a time window. Future studies should include more detailed measures of use.

Last, with our current design we cannot draw definite conclusions about causality, since we do not have a group of cannabis users that was cannabis-naïve at the start of the study. Moreover, our follow-up period did not cover early adolescence, the age range where cannabis may have a substantial impact on the developing brain [51]. Our findings can therefore be the result of (heavy) cannabis use before the start of our study or the result of the ongoing effect of a vulnerability factor that was already present before the start of the study or even before the onset of the use of cannabis.

## Conclusion

The lack of significant differences in GM volumes changes between young adult heavy cannabis users and healthy controls over time suggests that heavy cannabis use does not reduce regional GM volumes in this period. The cross-sectional negative correlations between medial temporal lobe volumes and the severity of cannabis use can either be a result of heavy cannabis use before early adulthood (with maximized damage before the onset of the current study) or represent a (genetic) vulnerability factor that was already present before the start of the study or even before the onset of the use of cannabis. Future studies should address these hypotheses in prospective designs in subjects that are drug naïve at the start of the study.

## Supporting Information

S1 Table **A. Overview of the findings of the baseline paper (Cousijn et al. 2012) and the current paper (Koenders et al., 2016).** Consistent findings are printed in bold. Note: CB = cannabis group; HC = healthy control group; CUDIT = Cannabis Use Disorder Identification Test; n.s. = not significant; x,y,z = MNI coordinates. **B. Overview of our results of the complete sample and of the control analyses.** Consistent findings are printed in bold. Note: CB = cannabis group; HC = healthy control group; CUDIT = Cannabis Use Disorder Identification Test; n.s. = not significant; x,y,z = MNI coordinates; a CB group N = 20, HC group N = 22; b CB group N = 14; HC group N = 20.
(DOCX)
