# Supplementary material for: Grey Matter Changes Associated with Heavy Cannabis Use: A Longitudinal sMRI Study
Source: PLoS One. 2016 May 25;11(5):e0152482. doi: 10.1371/journal.pone.0152482 (PMC4880314; doi:10.1371/journal.pone.0152482)
Supplement: S1 Table — A. Overview of the findings of the baseline paper (Cousijn et al. 2012) and the current paper (Koenders et al., 2016). Consistent findings are printed in bold. Note: CB = cannabis group; HC = healthy control group; CUDIT = Cannabis Use Disorder Identification Test; n.s. = not significant; x,y,z = MNI coordinates. B. Overview of our results of the complete sample and of the control analyses. Consistent findings are printed in bold. Note: CB = cannabis group; HC = healthy control group; CUDIT = Cannabis Use Disorder Identification Test; n.s. = not significant; x,y,z = MNI coordinates; a CB group N = 20, HC group N = 22; b CB group N = 14; HC group N = 20. (DOCX) [file pone.0152482.s001.docx]

# Supporting Information

**S1 Table A. Overview of the findings of the baseline paper (Cousijn et al. 2012) and the current paper (Koenders et al., 2016).**

|  | | | | Findings baseline paper (Cousijn et al., 2012). CB group N=33, HC group N=42 | | | | | Findings current sample (Koenders et al., 2016). CB group N=20; HC group N=22 | | | | |
| --- | --- | --- | --- | --- | --- | --- | --- | --- | --- | --- | --- | --- | --- |
|  |  |  |  | Cluster size | p _FWE cor_ | x | y | z | Cluster size | p _FWE cor_ | x | y | z |
| **BL** | group comparison | CB>HC | cerebellum | **4252** | **<.05** | **-8** | **-43** | **-20** | **393** | **.035** | **26** | **-70** | **-20** |
|  | Negative correlation | CUDIT | amygdala | **266** | **<.05** | **24** | **0** | **-24** | **8** | **.034** | **22** | **-4** | **-23** |
|  |  | Gram/ week | Hippocampus L | 361 | <.05 | -32 | -36 | -11 |  | n.s. |  |  |  |
|  |  |  | Hippocampus R | 345 | <.05 | 30 | -27 | -18 |  | n.s. |  |  |  |
| **FU** | Negative correlation | Gram/ week | Superior Temporal Gyrus |  | n.s. |  |  |  | 518 | .044 | -52 | 11 | -15 |
|  |  |  | amygdala |  | n.s. |  |  |  | 43 | .044 | -21 | -9 | -21 |
|  |  |  | hippocampus |  | n.s. |  |  |  | 118 | .044 | -27 | -10 | -20 |

Consistent findings are printed in bold.

## Note: CB=cannabis group; HC= healthy control group; CUDIT=Cannabis Use Disorder Identification Test; n.s.= not significant; x,y,z=MNI coordinates.

**S1 Table B. Overview of our results of the complete sample and of the control analyses.**

|  |  | **Baseline** | | **Follow up** | | |
| --- | --- | --- | --- | --- | --- | --- |
|  |  | group comparison | Negative correlation with CUDIT | Negative correlation with weekly amount of use (gram) | | |
|  |  | CB>HC |  |  |  |  |
|  |  | cerebellum | amygdala | Superior Temporal Gyrus | amygdala | hippocampus |
| Analysis in the complete sample (CB group, N=20, HC group, N=22). Covariates age and gender. | k | 393 | 8 | 518 | 43 | 118 |
|  | p _FWE cor_ | .035 | .034 | .044 | .018 | .044 |
|  | x, y , z | 26,-70,-20 | 22,-4,-23 | -52,11,-15 | -28,-9,-21 | -27,-10,-20 |
| Analysis in the complete sample. Covariates age, gender and AUDIT score as covariate | k | 230 |  | 450 | **28** | 75 |
|  | p _FWE cor_ | .094 | n.s. | .066 | **.023** | .074 |
|  | x, y , z | 26,-70,-20 |  | -52,11,-14 | **-28,-9,-21** | -28,-10,-21 |
| Analysis in the complete sample. Covariates age, gender and frequency of alcohol use as covariate | k | **466** | **3** | 438 | **31** | 78 |
|  | p _FWE cor_ | **.023** | **.039** | .073 | **.022** | .072 |
|  | x, y , z | **26,-58,-20** | **22,-4,-23** | -52,11,-15 | **-28,-9,-21** | -28,-10,-21 |
| Analysis in the complete sample. Covariates age, gender and FTQ score as covariate | k |  |  | 401 | **55** | **172** |
|  | p _FWE cor_ | n.s. | n.s. | .099 | **.015** | **.026** |
|  | x, y , z |  |  | -52,11,-14 | **-28,-9,-21** | **-27,-10,-21** |
| Analysis in the complete sample. Covariates age, gender and frequency of nicotine use (cigarettes/day) as covariate | k |  |  | 449 | **20** | 45 |
|  | p _FWE cor_ | n.s. | n.s. | .067 | **.027** | .114 |
|  | x, y , z |  |  | -52,11,-15 | **-28,-9,-21** | -28,-9,-21 |
| Analysis without subjects with polysubstance use or a comorbid psychiatric disorder (CB group, N=14, HC group, N=20) | k |  |  | 40 | **8** | 35 |
|  | p _FWE cor_ | n.s. | n.s. | .988 | **.039** | .142 |
|  | x, y , z |  |  | -52,11,-14 | **-28,-9,-18** | -32,-7,-18 |

Consistent findings are printed in bold.

## Note: CB=cannabis group; HC= healthy control group; CUDIT=Cannabis Use Disorder Identification Test; n.s.= not significant; x,y,z=MNI coordinates; ^a^ CB group N=20, HC group N=22; ^b^ CB group N=14; HC group N=20.
